# Attenuating Effect of Vigorous Physical Activity on the Risk for Inherited Obesity: A Study of 47,691 Runners

**DOI:** 10.1371/journal.pone.0031436

**Published:** 2012-02-23

**Authors:** Paul T. Williams

**Affiliations:** Life Sciences Division, Lawrence Berkeley National Laboratory, Donner Laboratory, Berkeley, California, United States of America; Fundación para la Prevención y el Control de las Enfermedades Crónicas No Transmisibles en América Latina (FunPRECAL), Argentina

## Abstract

**Objective:**

Physical activity has been shown to attenuate the effect of the FTO polymorphism on body weight, and the heritability of body weight in twin and in family studies. The dose-response relationship between activity and the risk for inherited obesity is not well known, particularly for higher doses of vigorous exercise. Such information is needed to best prescribe an exercise dose for obesity prevention in those at risk due to their family history.

**Design:**

We therefore analyzed self-reported usual running distance, body mass index (BMI), waist circumference, and mother's and father's adiposity (1 = lean, 2 = normal, 3 = overweight, and 4 = very overweight) from survey data collected on 33,480 male and 14,211 female runners. Age-, education-, and alcohol-adjusted regression analyses were used to estimate the contribution of parental adiposities to the BMI and waist circumferences in runners who ran an average of <3, 3–6, 6–9, ≥9 km/day.

**Results:**

BMI and waist circumferences of runners who ran <3 km/day were significantly related to their parents adiposity (P<10^−15^ and P<10^−11^, respectively). These relationships (i.e., kg/m^2^ or cm per increment in parental adiposity) diminished significantly with increasing running distance for both BMI (inheritance×exercise interaction, males: P<10^−10^; females: P<10^−5^) and waist circumference (inheritance×exercise interaction, males: P<10^−9^; females: P = 0.004). Compared to <3 km/day, the parental contribution to runners who averaged ≥9 km/day was diminished by 48% for male BMI, 58% for female BMI, 55% for male waist circumference, and 58% for female waist circumference. These results could not be attributed to self-selection.

**Conclusions:**

Exceeding the minimum exercise dose currently recommended for general health benefits (energy equivalent to running 2–3 km/day) may substantially diminish the risk for inherited obesity. The results are consistent with other research suggesting the physical activity dose required to prevent unhealthy weight gain is greater than that recommended for other health benefits.

## Introduction

As of 2008, 64% of U.S. adults have become overweight and 34% have become obese [1]. It is estimated that the health consequences of obesity represent ten percent of all medical expenditures and has an economic cost reaching $147 billion [2]. Abdominal visceral fat is specifically associated with multiple coronary artery disease risk factors including hypertension, insulin resistance, diabetes, and lipoprotein disorders, as well as coronary artery disease itself, and these relationships are independent of total body fat [3], [4]. Greater body weight and intra-abdominal fat significantly increase the risks for hypertension, diabetes, high cholesterol and coronary heart disease even within the ostensibly “healthy weight” range [5], [6]. To maintain healthy weight, sixty minutes of walking per day or its energy equivalent is recommended by the National Institute of Medicine (IOM) [7]. Sustained vigorous exercise, in particular, may inhibit age-related weight gain [8], [9].

Family and twin studies show that genetic factors account for 40% to 70% of the population variation in body mass index (BMI) [10], [11], however, the 32 single nucleotide polymorphisms (SNP) thus far achieving genome-wide significance for BMI account for only 1.45% of its population variance [12], with the largest portion (0.34%) due to SNPs associated with the fat mass and obesity (FTO) associated gene. The “missing heritability” of BMI and other complex traits is hypothesized to be due to unidentified rare and structural variants and gene-environment interactions [13].

One important gene-environment interaction affecting obesity involves physical activity. Several observations suggest that physical activity may mitigate the inheritance of body weight. In contrast to high heritability for BMI reported by others [10], [11], we reported that BMIs were unrelated in exercise-discordant monozygotic (MZ)-twin pairs whose running distances differed by 56 km/wk [14]. In that study, none of the active twins who had an overweight sedentary twin were themselves overweight [14]. These observations were consistent with structural equation modeling that showed that physical activity attenuated BMI inheritance in veteran Vietnam-Era twins [15]. Physical activity has been shown to mitigate the effects of the FTO genotypes on BMI [16]–[19]. Smaller weight gains over time have also been reported in the physically active compared to the sedentary members of 42 discordant twins studied prospectively [20].

While being physically active is known to produce multiple health benefits, official public health guidelines from government and scientific organizations do not currently recognize its importance in reducing the risk for inherited obesity. Moreover, except for our earlier study in walkers [21], the dose-response relationship between physical activity and mitigation of the risk for inherited obesity remains largely described. Establishing this benefit for running is important because: 1) running is a vigorous intensity physical activity because it requires >6-fold the energy expenditure of sitting at rest (>6 metabolic equivalents or 6 MET, where 1 MET = 3.5 ml O_2_•kg^−1^•min^−1^ [22]) whereas walking is classified as moderately intense because it expends only 3 to 6 METs [23]; and 2) total energy expenditure is usually greater for runners than walkers. Whereas our previous report showed that walking >4.5 km/day reduced the parental contribution to BMI by 36% vis-à-vis <1.5 km/day [21], the current analyses suggest running >9 km/day reduces the parental contribution to their BMI by 48% to 58% vis-à-vis <3 km/day, a substantial improvement. Because the energy required to walk 4.5 km/day (the highest exercise category in our previous study of walkers) is almost exactly equal to that of running 3 km/day (the lowest exercise category of the current report) [22], the previous and current reports are complementary in providing evidence for progressively greater reductions over a broad continuum of energy expenditures.

## Results

There were 51,697 participants with complete data on age, education, distance run, body mass index, and alcohol intake who did not smoke, of whom 4,006 were excluded because they did not provide information on their parents adiposity or stated that the information was unknown. Table 1 displays the men's and women's characteristics by distance run. The higher-mileage runners were younger, less educated if male, and drank slightly less. They were also significantly leaner, and perceived their mothers to be less overweight than lower-mileage runners. The runners reported that 16.7% of their mothers and 19.6% of their fathers were lean, 44.1% and 46.7% were average, respectively, 32.0% and 28.5% were overweight, respectively, and 7.2% and 5.2% were very overweight, respectively. The male runners age ranged from 18 to 86 years old in those who reporting <3 km/day, from 18 to 87 years in those reporting 3 to 6 km/day, from 18 to 82 in those reporting 6 to 9 km/day, and from 18 to 84 in those reporting ≥9 km/day. In females, the corresponding ranges were 18 to 85, 18 to 88, 18 to 79, and 18 to 72 years old, respectively.

**Table 1:** Characteristics (mean±SD) of runners by reported distance run per day.

	<3 km/day	3–6 km/day	6–9 km/day	≥9 km/day	Significance
**Male runners**					
Sample	7,783	14,447	6,291	4,959	
Age (years)	44.89±10.92	44.80±10.11	43.73±10.31	41.11±10.60	<10^−15^
Education (years)	16.55±2.46	16.50±2.42	16.39±2.50	16.25±2.54	<10^−12^
Alcohol (g/week)	77.46±108.62	84.34±116.86	80.78±114.68	72.80±111.04	0.003
Appearance Mother (1,…,4)	2.32±0.84	2.28±0.82	2.26±0.81	2.28±0.82	0.004
Appearance Father (1,…,4)	2.22±0.80	2.23±0.80	2.22±0.79	2.23±0.79	0.43
BMI (kg/m^2^)	24.96±2.99	24.07±2.49	23.31±2.24	22.57±2.20	<10^−15^
Waist circumference (cm)	87.03±7.04	84.93±5.81	82.94±5.33	80.95±5.14	<10^−15^
Chest circumference (cm)	103.63±7.73	102.29±7.20	100.84±6.97	99.32±7.26	<10^−15^
**Female runners**					
Sample	3,671	6,085	2,668	1,787	
Age (years)	38.73±10.15	38.61±9.90	37.35±9.86	36.31±9.77	<10^−15^
Education (years)	15.85±2.38	15.89±2.36	15.85±2.36	15.84±2.38	0.80
Alcohol (g/week)	49.08±75.39	48.86±71.51	48.29±71.91	42.46±74.89	0.005
Appearance Mother (1,…,4)	2.38±0.85	2.33±0.83	2.31±0.82	2.26±0.84	<10^−8^
Appearance Father (1,…,4)	2.16±0.83	2.12±0.82	2.12±0.80	2.09±0.83	0.008
BMI (kg/m^2^)	22.13±2.99	21.30±2.25	20.75±2.05	20.26±1.93	<10^−15^
Waist circumference (cm)	70.74±7.93	68.73±6.22	67.26±5.68	66.18±5.55	<10^−15^
Chest circumference (cm)	89.48±5.69	88.23±4.84	87.30±4.64	86.42±4.52	<10^−15^
Hip circumference (cm)	93.36±7.40	91.50±6.22	89.57±5.74	88.12±5.92	<10^−15^

Significance refers to the regression slope for the characteristic (dependent variable) vs. km/day run (independent variable). Data on waist circumference was provided by 92.9% of runners, chest circumference by 84% of runners, and hip circumference in 88.1% of female runners.

### Separate contributions of the mother and father to the runner's adiposity by running distance

Table 2 presents the regression slopes for the runners' BMIs and body circumferences (dependent variable) vs. the mothers' and fathers' adiposity (independent variable) adjusted for the runners' age, education, and reported alcohol intake. Among runners averaging <3 km/day, BMI and circumferences of the waist, hip and chest were all strongly related to their parents' adiposity. The contribution of the parents' adiposities to the runners' BMIs and waist circumferences (i.e., kg/m^2^ or cm per increment in parental adiposity) diminished significantly with running distance, e.g., only about one-half as great for runners averaging >9 km/day vis-à-vis ≤3 km/day. The mothers' contributions to the runners' chest circumferences also diminish with increasing running mileage, whereas attenuation of the fathers' contributions with increased running mileage was less significant. Hip circumference in female runners remained consistently related to their parent's adiposity levels regardless of running mileage.

**Table 2:** Regression slopes (±SE) for body mass index (BMI) and body circumferences vs. reported obesity status of the subject's mother and father (kg/m^2^ or cm per increment) adjusted for age, education, and alcohol intake and stratified by the subject's running distance.

	Dependent variable			
	BMI (kg/m^2^)	Waist circum-ference (cm)	Chest circum-ference (cm)	Hip circum-ference (cm)
**Mother's contribution**				
*Male runners*				
<3 km/day	0.55±0.04§	1.03±0.09§	0.94±0.11§	
3–6 km/day	0.37±0.02§	0.60±0.06§	0.74±0.08§	
6–9 km/day	0.34±0.03§	0.56±0.08§	0.60±0.12§	
≥9 km/day	0.29±0.04§	0.52±0.09§	0.66±0.14§	
*Interaction*	*P<10^−8^*	*P<10^−6^*	*P = 0.003*	
*Female runners*				
<3 km/day	0.49±0.06§	0.95±0.16§	0.51±0.12§	0.89±0.15§
3–6 km/day	0.27±0.03§	0.49±0.10§	0.25±0.08‡	0.66±0.10§
6–9 km/day	0.30±0.05§	0.45±0.14‡	0.03±0.12	0.66±0.14§
≥9 km/day	0.27±0.05§	0.56±0.17‡	0.39±0.13†	0.95±0.17§
*Interaction*	*P = 0.0003*	*P = 0.02*	*P = 0.06*	*P = 0.51*
**Father's contribution**				
*Male runners*				
<3 km/day	0.39±0.04§	0.77±0.10§	0.64±0.12§	
3–6 km/day	0.27±0.03§	0.40±0.06§	0.46±0.08§	
6–9 km/day	0.22±0.04§	0.33±0.08§	0.25±0.12*	
≥9 km/day	0.19±0.04§	0.23±0.09†	0.28±0.14*	
*Interaction*	*P = 0.0001*	*P = 0.0001*	*P = 0.04*	
*Female runners*				
<3 km/day	0.42±0.06§	0.73±0.16§	0.39±0.12‡	0.62±0.15§
3–6 km/day	0.21±0.03§	0.33±0.10‡	0.24±0.08†	0.45±0.10§
6–9 km/day	0.24±0.04§	0.31±0.15*	0.35±0.12†	0.55±0.15‡
≥9 km/day	0.12±0.05*	0.14±0.17	0.30±0.13*	0.51±0.18†
*Interaction*	*P = 0.002*	*P = 0.10*	*P = 0.96*	*P = 0.62*

Adjusted for age, education, and alcohol intake. Significance levels coded:

*P<0.05,

† P<0.01,

‡ P<0.001,

§ P<0.0001.

### Joint contributions of the mother and father to the runners adiposity by running distance

Multiple regression analyses were used to measure the independent contributions of the mothers' and fathers' adiposities to the runners' BMIs and body circumferences (Table 3). Among the low-mileage offspring, both parent's contributed independently to the runner's values. In male runners, the mothers' contributions were significantly greater than the fathers' for BMI (P = 0.001), waist circumference (P = 0.03), and chest circumference (P = 0.05, analyses not displayed). In female runners, these differences did not achieve statistical significance (P = 0.32, P = 0.31, P = 0.40, respectively), which may be due, in part, to their smaller sample size.

**Table 3:** Multivariate regression to determine the linear combinations of reported obesity of the mother and father that best predicts body mass index (BMI) and body circumferences in runners who averaged <3 km/day.

	Slopes ±SE	
	Mother's contribution	Father's contribution
*Male runners*		
BMI (kg/m^2^)	0.51±0.04§	0.32±0.04§
Waist circumference (cm)	0.96±0.09§	0.65±0.10§
Chest circumference (cm)	0.87±0.11§	0.52±0.12§
*Female runners*		
BMI (kg/m^2^)	0.45±0.06§	0.37±0.06§
Waist circumference (cm)	0.88±0.16§	0.63±0.16§
Chest circumference (cm)	0.48±0.12§	0.33±0.12†
Hip circumference (cm)	0.83±0.15§	0.53±0.15‡

Adjusted for age, education, and alcohol intake. Significance levels coded:

*P<0.05,

† P<0.01,

‡ P<0.001,

§ P<0.0001.

**Figure 1:**
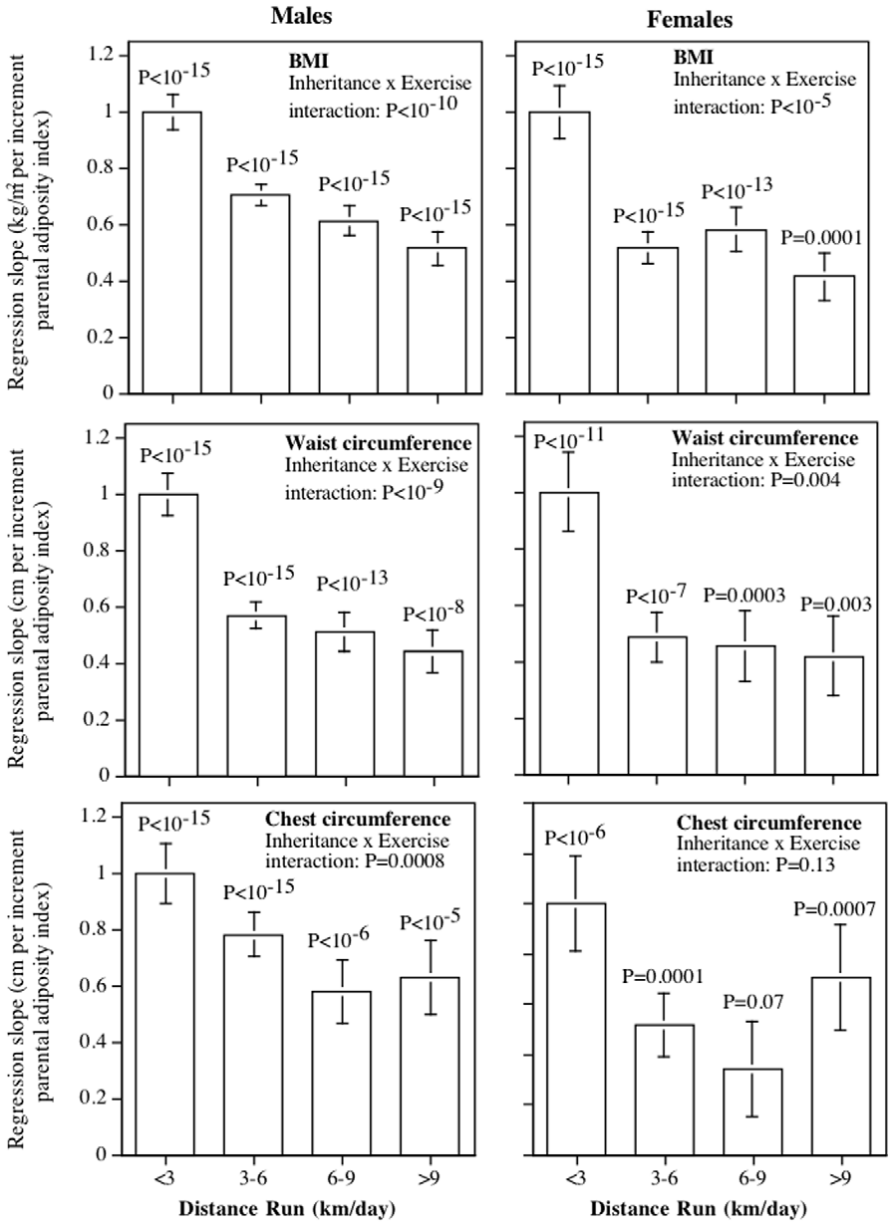
Reduced impact of parental adiposity as a risk factor for excess body weight with greater distance run. The parental adiposity index is the linear combination of the mothers' and fathers' adiposities that most strongly predicted the runners' adiposity within the <3 km/day group. In the analyses, the coefficient (slope) for the <3 km/day category is always one because it was the category of runners used to define the index. Significance levels above each bar refer to the significance of the parent-offspring relationship within each distance category. The inheritance x exercise interaction tests whether the parent-offspring relationships differed by the offsprings' running distance. When the coefficients (slopes) for other running categories (3–6, 6–9, ≥9 km/day) are less than one, this means that exercise has attenuated the parental contribution. Specifically, the coefficient estimates the reduction in the effect of the parents' adiposities on their offsprings' BMIs and body circumferences.

The coefficients of Table 3 define the best linear combination of the parents' adiposity for predicting the runners' BMI and body circumferences, and were used to define the combined “parental adiposity index”. For example, the Table shows that “0.51*mother's adiposity +0.32*father's adiposity” was the best predictor of a male runner's BMI, and that “0.45*mother's adiposity +0.37*father's adiposity” the best predictor of a female runner's BMI. Separate parental adiposity indices were computed for male and female runners, however, the same index (i.e., derived from the <3 km/day group) was applied to all running categories in producing the bar graph of Figure 1. The analyses are exactly the same as those presented in Table 2, except that the parental adiposity index replaces of mother's and father's adiposities as independent variables. In the analyses, the coefficient (slope) for the <3 km/day category is always one because it was the category of runners used to define the index. When the coefficients (slopes) for other running categories (3–6, 6–9, ≥9 km/day) are less than one, this means that exercise has attenuated the parental contribution. Specifically, the coefficient shows the degree that exercise reduces the contribution of the parents' adiposities to the runners' BMIs and body circumferences.

Figure 1 shows that the parental contribution to male runners' BMIs was reduced by 29% in runners who ran 3–6 km/day, 39% for those who ran 6–9 km/day, and 48% for runners who exceeded 9 km/day. This represented a highly significant overall decline in the inheritance of BMI with increasing exercise (P<10^−10^). More specifically, the parental contribution was significantly less for male runners who ran ≥3 than <3 km/day (P<10^−15^, not displayed in the figure), and for male runners who ran ≥6 than 3 to 6 km/day (P = 10^−8^), but not between those who ran >9 vs. 6 to 9 km/day (P = 0.85). Among female runners, the parental contribution was significantly less for those who ran ≥3 than <3 km/day (P<10^−13^), but the parental contribution did not differ significantly for those who ran ≥6 than 3 to 6 km/day (P = 0.45) or ≥9 vs. 6 to 9 km/d (P = 0.21). Thus a significant progressive dose-response relationship exists between the dose of vigorous exercise and the inheritance of parental adiposity through at least 6 km/day in men and 3 km/day in women.

Figure 1 also suggests that exercise attenuated the parental contribution to regional adiposity in a dose-dependent manner for both male and female offspring. Specifically, the parental contribution to the runners' waist circumferences decreased significantly with mileage in both men (P<10^−9^) and women (P = 0.004). More detailed comparisons (not displayed) showed that the parental contribution was significantly reduced in runners who ran >3 vs. ≤3 km/day (males: P<10^−15^; females: P<10^−8^), and in male but not female runners who ran >6 vs. 3 to 6 km/day (males: P<10^−9^; females: P = 0.15). The parental contribution to the runners' chest circumferences also decreased significantly with running mileage for male runners (P<10^−9^), with a weaker contribution for those ≥3 vs. <3 km/day (P = 0.0002, not displayed), and ≥6 vs. 3–6 km/day (P = 0.0006), and no further attenuation thereafter. Although the parental contribution to female chest circumference did not decline linearly with running mileage (P = 0.3), the parental contribution for those who ran ≥3 km/day was significantly weaker than in those who ran <3 km/day (P<0.0001).

The above results were based on the particular linear combinations showing the greatest parental contribution to the least active runners' BMIs and body circumferences (Table 3). Similar results were obtained using the simple average of the mothers' and fathers' adiposities, i.e., the attenuation of the parental contribution with mileage was P<10^−9^ and P<10^−5^ for the male and female runners' BMIs, respectively, P<10^−9^ and P = 0.005 for the male and female runners' waist circumferences, respectively, and P = 0.0009 and P = 0.18 for the male and female runners' chest circumferences, respectively (analyses not displayed).

### Ruling out self-selection

**Figure 2:**
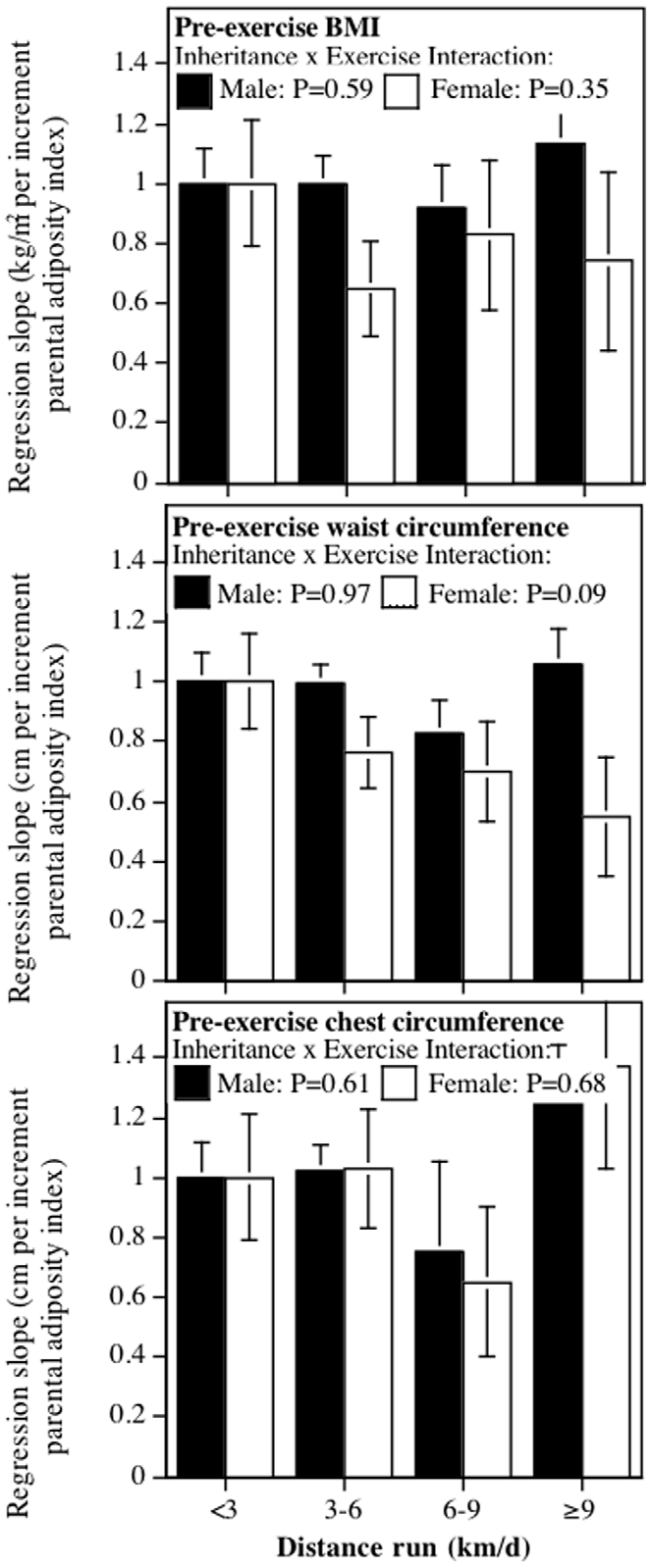
Effect of parental adiposity on runners' pre-exercise BMI, waist circumference, and chest circumference, showing no significant association with exercise. The inheritance x exercise interaction tests whether the parent-offspring relationships differed by the offsprings' running distance. Parental adiposity was strongly related to pre-exercise BMI in male runners (P<10^−15^ all categories) and female runners who currently ran <3 (P<10^−15^), 3–6 and (P<10^−15^), 6–9 (P<10^−11^), and >9 km/day (P<10^−7^), pre-exercise waist circumference in male and female runners who currently ran <3 (P<10^−15^ and P<10^−9^, respectively), 3–6 (P<10^−15^ and P<10^−10^, respectively), 6–9 (P<10^−13^ and P<0.0001, respectively), and ≥9 km/day (P<10^−15^ and P = 0.006, respectively), and pre-exercise chest circumference in male and female runners who currently ran <3 (P<10^−15^ and P = 0.0003, respectively), 3–6 (P<10^−15^ and P<10^−6^, respectively), 6–9 (P<10^−6^ and P = 0.03, respectively), and ≥9 km/day (P<10^−12^ and P<10^−4^, respectively). The analyses are restricted to the 96.7% of men and 94.3% of women who provided a weight for when they had first started running 12 or more miles per week.

To test whether self-selection explained the weaker parental contribution to high-mileage runners, the analyses of Figure 1 were repeated using the participants' recalled age and weight, waist circumference, and chest circumference when they first started running 12 or more miles per week (pre-exercise BMI, pre-exercise waist, and pre-exercise chest circumference). The analyses, Figure 2, necessarily exclude 3.3% of men and 5.7% of women who did not provide these data, presumably because they had never run at least 12 miles per week. In contrast to the highly significant interaction terms of Figure 1, current running distance was not significantly related to the effect of parental adiposity on the runners' pre-exercise BMI (male: P = 0.59, female: P = 0.35), pre-exercise waist circumference (male: P = 0.97, female: P = 0.09), or pre-exercise chest circumference (male: P = 0.61, female: P = 0.68). Parental adiposity was significantly related to pre-exercise BMI and waist and chest circumferences within each distance category, however.

## Discussion

The novel findings from these analyses are the strong dose-response relationships between the amount of vigorous physical activity performed and decreased inheritance of parental adiposity, that are substantially greater than can be attributed to chance (Table 2, Figure 1). The attenuation was similar in men and women, albeit less significant in women presumably because there were only about one-quarter as many women as men. The attenuated relationships included not only total adiposity as measured by BMI, but also regional adiposity as measured by circumferences of the waist and chest. Waist circumference reflects abdominal visceral adipose tissue accumulation [24], and chest circumference is a measure of upper body obesity that exhibits relationships to plasma leptin levels that are not apparent for waist or hip measurements [25]. Thoracic fat has also been related to low-density lipoprotein levels [26]. Figure 2 shows that our results are unlikely to be due to runners who are immune to their parent's adiposities choosing to run longer distances. Specifically, distance run did not change the relationship between parents' adiposity and the runners' pre-exercise BMI, pre-exercise waist circumference, and pre-exercise chest circumference. Previous analyses of this cohort showed that self-selection for running distance based on pre-exercise BMI explained only 17% of BMI in men, albeit 40% in women [27].

Compared to <3 km/day, the parental contribution to runners who averaged >9 km/day was diminished by 48% for male BMI, 58% for female BMI, 55% for male waist circumference, and 58% for female waist circumference. These reductions are substantially greater than the cumulative effect of all 32 SNPs currently identified as having confirmed genome-wide significance with BMI [12]. Thus, our ability to manipulate genetic risk for obesity exceeds our understanding of the genetics themselves.

Physical activity recommendations are prescribed in units of METmin/week. This is a measure of cumulative total activity as calculated from the products of the MET value for each aerobic (endurance) physical activity and the time spent per week performing that activity. The American Heart Association and the American College of Sports Medicine recommend that all healthy adults aged 18 to 65 years old perform a minimum of 450–750 METmin/week. The least active category of runners in the current analyses, those running <3 km/day, expended an average of 757 METmin/wk if male and 738 METmin/wk if female [23], which falls at the upper limit of the minimum recommended dose. Thus, these analyses suggest yet another important health benefit to exceeding the minimum guideline activity level [23] (which correspond more closely to the physical activity recommendations for weight maintenance by the Institute of Medicine [7]).

The current analyses extend our previous findings in walkers, which showed that compared to the most sedentary walkers [21], the effect of parental adiposity on offspring BMI was reduced 36% for offspring who exceeded 4.5 km/day vis-à-vis 1.5 km/day. Walking may be more attractive and readily adopted than running by overweight and obese individuals who find the prospect of vigorous physical activity daunting [28]. The current results provide evidence for more substantial immunity from parental obesity for those who are able to exercise more intensely and for longer durations. These effects may be evidence of a more general phenomenon of obesity risk factors having a greater effect on overweight and obese men and women than those who are lean [29]. In this regard, we have also demonstrated that diet appeared to produce less effect in the generally leaner high-mileage runners and walkers vis-à-vis the generally heavier low-mileage runners and walkers [30], [31].

It is commonly acknowledged that exercise per se is not an effective means for achieving substantial weight loss, but plays an essential role in weight maintenance [7]. It is our opinion that the true value of exercise in addressing the obesity epidemic is in primary prevention [8], [9]. Various mechanisms may contribute to the prophylactic effect of exercise against the risks of inherited weight gain. Running promotes weight loss [9], and attenuates age-related weight gain in proportion to the exercise dose [8]. These results may be due to improved fat oxidation [32] and improved coupling between energy intake and expenditure [33]. Sex steroid and growth hormone deficiencies explain, in part, the accumulation of visceral fat with aging [34], [35]. Visceral adipocytes are distinguished from subcutaneous adipocytes in their increased sensitivity to lipolytic stimuli and decreased sensitivity to the antilipolytic effect of insulin [34]. The concentration of specific hormone receptors, blood flow, and innervation are all greater in visceral fat than in other fat depots [34]. Androgen receptor density, in particular, is higher in visceral than other adipose tissue, and the receptors are up regulated by testosterone [34]. An inverse relationship between greater running distance and waist circumference is well established (Table 1), as is the heritability of regional fat distribution as measured by waist to hip ratio (22% to 68% [36]). Figure 1 shows that the effects of exercise and parental inheritance are not additive for either BMI or waist circumference, but rather involve a diminished parent-offspring concordance at higher activity levels. In contrast, hip circumference in women decreased significantly with running distance, and there was a strong concordance between a women's hip circumference and her parent's adiposity, but the effects were additive (i.e., no significant interaction). This difference between waist (visceral) and hip (gluteal) fat patterns may relate to intrinsic differences in the metabolism and regulation of the fats. In addition, genetic predisposition to female fat patterns may be protected as a consequence of sexual selection bestowing reproductive advantage, at least evolutionarily.

Finally, we note that these analyses confirm that parental adiposity was a significant risk factor for greater BMI and circumferences of the waist, hip, and chest. Despite the nonspecificity of the parental adiposity question, the analyses demonstrated that parental adiposity significantly predicted the runners' BMIs (both current and pre-exercise) and waist circumferences (both current and pre-exercise) in eight out of eight subsets, and the runners' chest circumferences in at least seven of the eight subsets (both current and pre-exercise). The associations were significant when looking at paternal and maternal adiposity separately, and in both male and female offspring, and are consistent with the substantial heritability reported for twin and family studies [10], [11]. The mechanism by which parental adiposity affects total and regional adiposity of the runners may not be exclusively genetic, and may include effects due to family environment. For example, families where one or both parents are overweight reportedly have children who snack more frequently on energy-dense snacks [37], and studies have shown significant correlations between parent's and offspring's dietary intake [38] and food preferences [39].

### Limitations

The primary limitation of these analyses is that the presumed effect of running on the inheritance of body weight is inferred from their cross-sectional association rather than an effect of exercise training. Limited dietary data were available in these runners, therefore conclusions regarding increased running distance inhibiting one's genetic predisposition for being overweight may be in part due to more active individuals leading healthier lifestyles and therefore more likely to expend, and less likely to consume, excess calories. In this regard, it should also be acknowledged that the IOM recommendations are also primarily derived from cross-sectional associations between energy metabolism and BMI [7]. We also acknowledge the limitation of the runners' subjective classification of their parents as lean, average, overweight and very overweight. However, greater measurement error would diminish parent-offspring concordance and would not give rise to diminished concordance with increasing exercise unless the misclassification was substantially greater for higher mileage than lower mileage runners [40]. Moreover, if the significant interactions of figure 1 were due entirely to a systematic bias in the perception of the parent's adiposity, then presumably this would also affect the associations for the runners' pre-exercise weights and body circumferences (Figure 2), which were not observed. Body circumferences were obtained by self-report. The relationships between circumferences and running distances could be weakened by different locations of where waist, hip and chest circumferences were measured. However, unless the perceived location varied systematically in relation to running distance, the subjectivity is unlikely to produce the relationships reported in the tables and figures. Finally, we acknowledge that runners may not be necessarily representative of the general population. Specifically, the runners reported that only 39.2% of their mothers and 33.7% of their fathers were overweight or very overweight, which is less than the general population [1]. However, we do not expect that the metabolic processes affecting body weight in runners are fundamentally different than those of nonrunners. Running has the advantage of its energy expenditure being estimated as a simple function of distance run rather than the cumulative products of duration and intensity over a variety of physical activities [22]. Vigorous-intensity activities, such as running, are generally more accurately reported than moderate- or light-intensity activities [41], and this improves the signal to noise ratio and increases statistical power. This may explain, in part, our success in demonstrating that running predicts weight gain prospectively [8] while physical activity measured in the general population usually does not [42].

In conclusion, these analyses demonstrate an additional health benefit of higher doses of vigorous exercise, i.e. providing a prophylactic against the inherited risk for weight gain. They also likely demonstrate substantial gene-environment interactions involving physical activity and the genetics of obesity. The risks of many chronic diseases, including hypertension, hypercholesterolemia, and diabetes become almost negligible in the lower portion of the healthy weight range [5], [6]. Exercise may not only provide a prophylactic for the risks of inherited weight, but also its associated health consequences.

## Materials and Methods

The National Runners' Health Study has been described in numerous publications [5], [6], [8], [9], [21], [27], [29], [30], [43], [44], [45]. A two-page mailed questionnaire, sent to subscribers of a running magazine and to participants of running events, solicited information on demographics (age, race, education), running history (age when began running at least 12 miles per week, current average weekly mileage, number of marathons run over the preceding 5 years, best marathon and 10-km race times), weight history (greatest and current weight; weight when started running; least weight as a runner; body circumferences of the chest, waist, and hips; bra cup size), diet (vegetarianism and the current weekly intakes of alcohol, red meat, fish, fruit, vitamin C, vitamin E, and aspirin), current and past cigarette use, history of heart attacks and cancer, and medications for blood pressure, thyroid, cholesterol, or diabetes [43], [44]. Running distances were reported in miles per week, body circumferences in inches, and body weights in pounds. These values were converted to kilometers per day, centimeters, and kilograms, respectively. The test-retest correlations for self-reported distance run per week (r = 0.89) [45] compares favorably with those reported by others. A four point scale of the mother's and father's adiposity was assessed from the question: Would you describe your mother (father) as: 1) lean, 2) average, 3) overweight, 4) very overweight, 5) unknown.

The runners' BMIs were calculated as the weight in kilograms divided by height in meters squared. Self-reported body circumferences of the waist, hip, and chest were in response to the question “Please provide, to the best of your ability, your body circumferences in inches” without further instruction. Self-reported height and weight from the questionnaire have been found previously to correlate strongly with their clinic measurements (r = 0.96 for both) [45]. Self-reported waist circumferences are somewhat less precise as indicated by their correlations with self-reported circumferences on a second questionnaire (r = 0.84) and with their clinic measurements (r = 0.68) [45]. Self-reported chest circumferences also demonstrate strong test-retest correlations across repeated questionnaires (r = 0.93) and somewhat weaker correlation relative to their clinic measurement (r = 0.77) [45]. The protocol for this study was reviewed and approved by the University of California Berkeley committee for the protection of human subjects, and all subjects provided a signed a statement of informed consent.

### Statistical analyses

Results are presented as mean±SE or slopes±SE except where noted. With the exception of the sample description of Table 1, all analyses were adjusted for age (age and age^2^), education, and alcohol intake. Multiple regression analyses were used to test whether the mothers' and fathers' adiposity affected the runners' BMI and body circumferences. Specifically, we tested whether the coefficient for the interaction between parent's adiposity x distance run differed significantly from zero in a model that also included the separate effects of the parent's adiposity and distance run. In these analyses parental adiposity was defined as the mother's adiposity alone, the father's adiposity alone, the average of the mother's and father's adiposity, or a combined parental adiposity index. The combined “parental adiposity index” was defined as the best linear combination of the mother's and father's adiposity for predicting offspring BMI or body circumferences as determined by standard linear regression within the least active running category (i.e., within the <3 km/day category). We also divided the sample into running increments of 0–3, 3–6, 6–9, and >9 km/day and calculated the regression coefficients for parental adiposity (mother, father, average, or parental adiposity index) separately within each stratum. The significance levels for incremental reductions in the slope of the parent's adiposity were computed as follows: we included a single coefficient for parental adiposity for all subjects, and then tested the significance when a separate coefficient was added for runners who ran <3 km/day (its significance implying that the slope for running ≥3 km/day was significantly less than <3 km/day). The analyses were then repeated including separate coefficients for both <3 km/day and 3–6 km/day (the significance of the 3–6 km/day coefficient implying that the slope for running >6 km/day was significantly less than 3–6 km/day), and separate coefficients for <3 km/day, 3–6 km/day, and 6–9 km/day (the significance of the 6–9 km/day coefficient implying that the slope for running >9 km/day was significantly less than 6–9 km/day).
